# Using structural phase transitions to enhance the coercivity of ferromagnetic films

**DOI:** 10.1063/1.5118893

**Published:** 2020-10

**Authors:** Ryan F. Need, Josh Lauzier, Logan Sutton, Brian J. Kirby, Jose de la Venta

**Affiliations:** 1 NIST Center for Neutron Research, National Institute of Standards and Technology, Gaithersburg, Maryland 20899, USA; 2 Department of Materials Science and Engineering, University of Florida, Gainesville, Florida 32611, USA; 3 Department of Physics, Colorado State University, Fort Collins, Colorado 80523, USA

## Abstract

Storing information in magnetic recording technologies requires careful optimization of the recording media’s magnetic properties. For example, heat-assisted magnetic recording (HAMR) relies on a prerecording heating step that momentarily lowers the coercivity of the ferromagnetic recording media, and thereby decreases the energy expenditure for each writing operation. However, this process currently requires local temperature increases of several hundred Kelvins, which in turn can cause heat spreading, damage the write head, and limit recording rates. Here, we describe a general mechanism for dramatically tuning the coercivity of ferromagnetic films over small temperature ranges, by coupling them to an adjacent layer that undergoes a structural phase transition w ith large volume changes. The method is demonstrated in Ni/FeRh bilayers where the Ni layer was deposited at 300 K and 523 K, above and below the FeRh metamagnetic transition at 370 K. When the Ni layer is grown at high temperatures, the 1% FeRh lattice expansion relative to room temperature alters the Ni’s crystallographic texture during growth and leads to a 500% increase in coercivity upon cooling through the FeRh’s metamagnetic transition. Our analysis suggests this effect is related to domain wall pinning across grain boundaries with different orientations and strain states. This work highlights the promise of thermally tuning the coercivity of ferromagnetic materials through structural coupling to underlying films that could enable simplified heatsink designs and expand the selection of materials compatible with HAMR.

## INTRODUCTION

Engineering the coercivity of magnetic materials is critical to improving the stability and efficiency of magnetic recording media.^1–4^ A central trade-off is that while larger coercive fields provide increased protection of information against thermal fluctuations and demagnetizing fields, they also increase the energy required to switch a bit. One rapidly developing alternative to this coercivity compromise is known as heat assisted magnetic recording (HAMR), in which the magnetic media undergoes local heating to briefly lower the coercivity immediately prior to each writing operation.^5–7^ However, reducing the coercivity of common magnetic storage media can require local temperature increases of several hundred Kelvins.^6,7^ In turn, these large heat loads create problems confining the heat laterally to enable high areal bit density, removing the heat quickly to achieve a rapid thermal response and recording rate, and protecting the write head from thermal degradation over time.^8–10^ While it is possible to address these issues individually through improved heatsink designs or better barrier coatings, finding new ways to modify magnetic coercivity and lower switching fields can circumvent these problems altogether.^11,12^ Particularly attractive are methods to increase a material’s thermal coercivity response [i.e., H_C_(T)], which can reduce the heat load required and directly address the source of HAMR’s heat management issue.

In this work, we demonstrate a thin film engineering approach to tune the coercivity of Ni/FeRh bilayers by 500% over just a 50 K temperature range. To accomplish this, we coupled the Ni layers to metamagnetic FeRh films. FeRh near its equiatomic composition crystallizes in the B2 CsCl-type structure and displays a peculiar first-order metamagnetic phase transition near 370 K.^13–18^ Below the metamagnetic transition, FeRh is a G-type antiferromagnet (AFM) with a 3.2 *μ*_B_ Fe moment.^19,20^ Above the transition, FeRh becomes ferromagnetic (FM) and acquires a Rh moment of 1 *μ*_B_.^20,21^ In addition, the magnetic transition is accompanied by a 1% isotropic lattice expansion,^22,23^ along with large changes in resistivity,^13,24^ magnetoresistance,^25,26^ and entropy.^27^ We leverage this lattice expansion during the FeRh transition to modify the coercivity in Ni layers grown at temperatures above and below the metamagnetic transition. Our analysis suggests that this coercivity enhancement is a general phenomenon of FM films structurally coupled to materials with first-order phase transitions, and therefore may provide an opportunity to tailor the properties of numerous technologically relevant magnetic materials.

## EXPERIMENTAL METHODS

Our samples were grown by depositing 70 nm of FeRh on top of MgO (001) substrates using DC sputtering from a target composition of Fe_50_Rh_50_. The deposition temperature was fixed at 573 K (300 °C), and the base pressure of the chamber was 1 × 10^−10^ bars (1 × 10^−7^ Torr). During the sputtering process, a 20 W power and an Ar pressure of 5 × 10^−3^ mbar (4 mTorr) were used. After FeRh deposition, the sample was annealed *in situ* at 1073 K (800 °C) for 2 h to promote the ordering of the B2 phase. Subsequently, a 15 nm Ni film was deposited on top of the FeRh layer at two different temperatures: 300 K (room temperature, RT) and 523 K (high temperature, HT). Finally, a 7 nm capping layer of tungsten was added at room temperature to prevent the oxidization of the Ni film.

X-ray diffraction (XRD) measurements were done in ambient conditions with a Rigaku SmartLab X-ray diffractometer equipped with a Cu Kα source.^28^ Magnetic measurements were performed on a Quantum Design Vibrating Sample Magnetometer (VSM). The following protocol was used for all magnetometry measurements: first the samples were heated to 450 K, then a magnetic field of μ_0_H = 1 T was applied to fully saturate the sample, and finally the data were recorded. Magnetization vs temperature measurements were collected under a 100 mT applied field by first cooling then heating the samples at 1 K/min rate. Magnetization vs field hysteresis loops were collected consecutively upon cooling from the high temperature FM phase (i.e., without reheating between loops).

Specular polarized neutron reflectometry (PNR) measurements were performed at the NIST Center for Neutron Research on the polarized beam reflectometer instrument using a monochromatic (4.75 Å) and polarized neutron beam.^29^ For direct comparison with the VSM measurements, the sample was heated in zero field to 450 K, saturated under a μ_0_H = 700 mT in-plane field, then the field was reduced to 100 mT, and the temperature brought down to 350 K. An initial PNR scan was collected at +100 mT, then the field was cycled through negative saturation (−700 mT) and back to +25 mT. The PNR data were reduced using the online reductus package and analyzed in the Refl1d package.^30,31^ A single nuclear scattering length density (SLD) model was corefined along with two magnetic SLD models to the 100 mT and 25 mT datasets. This ensures the physical requirement that the two refined magnetic depth profiles correspond to the same chemical sample structure (see the supplementary material).

## RESULTS

**FIG. 1.**
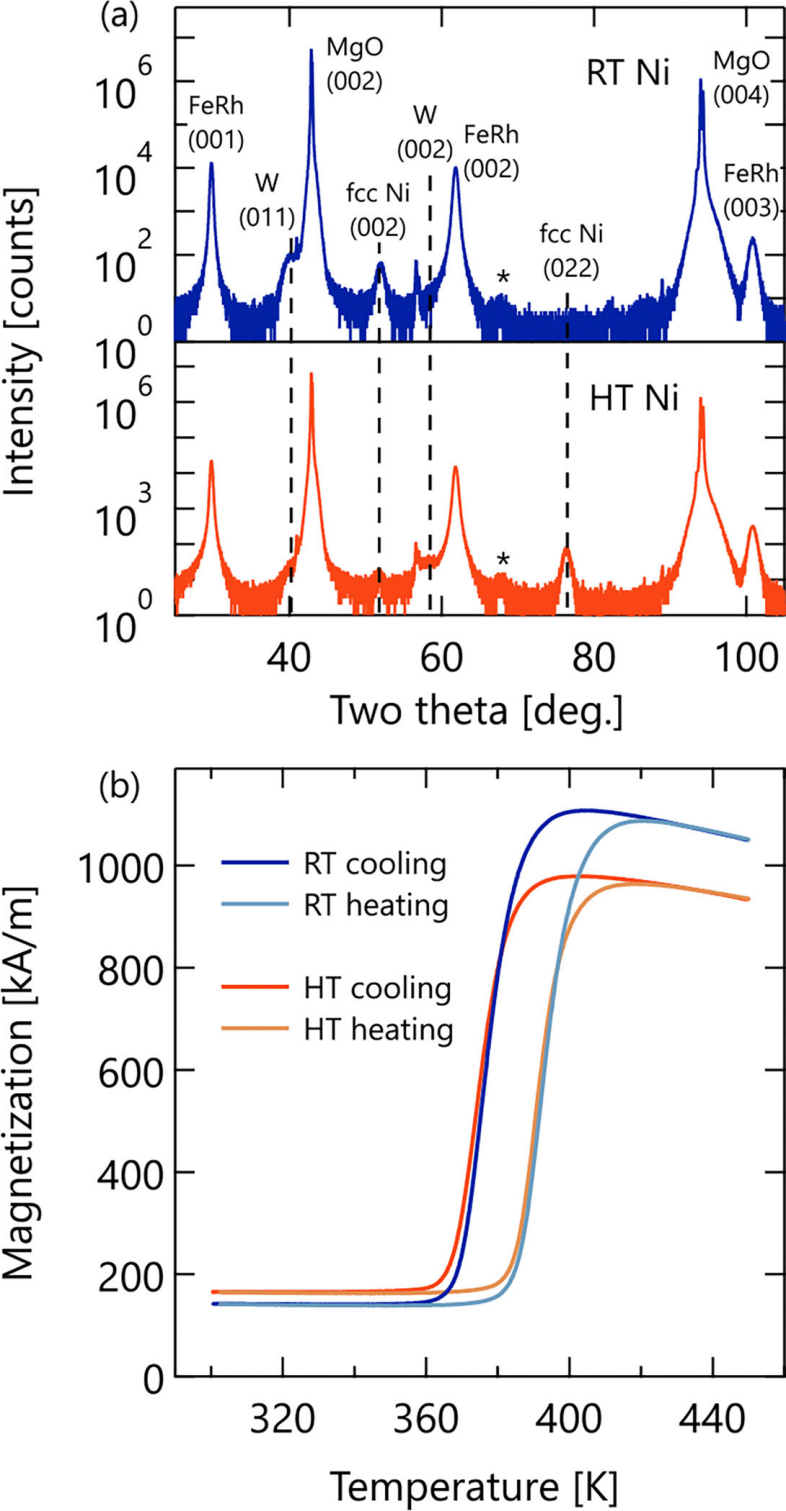
(a) Out-of-plane Cu Kα X-ray diffraction pattern collected at room temperature, comparing the RT Ni sample (top, blue) to the HT Ni sample (bottom, red). Asterisks mark an Al powder line from the sample holder. (b) In-plane magnetization as a function of temperature under a 100 mT applied field.

Figure 1 compares the growth, crystallinity, and chemical and magnetic order parameters of the two samples. Considering first the FeRh films, the XRD shown in Fig. 1(a) indicates that both films grew along the [00*L*] direction, parallel to MgO [00*L*], as expected given the growth conditions used.^15–17^ Crystalline quality of the FeRh films was analyzed using the FeRh (001) full-width half maximum where both samples had a near identical value of 0.318(1)°. From the position of the FeRh (00*L*) reflections, we determine the c-axis lattice constant of both samples to be 3.000(1) Å, in excellent agreement with prior values reported for thin films.^15–17^ Furthermore, as the magnetic anisotropy of FeRh films is largely controlled by the c/a ratio, the shared lattice constants also indicate nearly identical magnetic anisotropy.^17^ Finally, by comparing the area of the FeRh (001) and (002) reflections, the chemical ordering of the FeRh B2 phase can be described by an order parameter, *S*, calculated as *S* ≈ [(*A*_001_/*A*_002_)/1.07]^1/2^.^24^ Here, we see a slight difference between the two films, with the FeRh in the HT sample possessing slightly a higher order, *S*_*HT*_ = 0.866(1), than the FeRh in the RT sample, *S*_*RT*_ = 0.810(1). This may reflect a slight difference in FeRh stoichiometry or annealing as is discussed in more detail below.^17,24^

Magnetically, the FeRh layers are also quite similar to one another as shown in Fig. 1(b), which plots the in-plane magnetization behavior of the FeRh bilayers as a function of temperature. Given the relative thicknesses of the FeRh and Ni layers (≈5:1), it is the FeRh magnetic transition from the high temperature FM phase to the low temperature AFM phase that dominates the observed magnetization. Below 360 K, the magnetization is primarily that of the Ni film. We define the metamagnetic transition temperature (T_T_) as the temperature at which the magnetization value is half of its saturated value (M_S_), and determine it to be 374 K for both samples. As with its lattice parameters, T_T_ of FeRh is highly dependent on film stoichiometry,^16–18^ and thus, the shared transition temperature for both films once again suggests very similar FeRh films in the two samples.

There is however a notable difference (12%) in the M_S_ between the two samples, namely, M_S_ (400 K, cooling) = 1105 kA/m (RT) and M_S_ = 978 kA/m (HT). This difference in M_S_, along with the small difference in B2 chemical ordering, can best be explained by a slight composition difference between the two films, on the order of 1% or less, which on the Rh-rich side of the phase diagram can reduce M_S_ without notably altering the transition temperature.^32^ Note that a difference in FeRh thickness could, in principle, lead to the difference in M_S_; however, X-ray reflectivity measurements determined the FeRh layers to be the same thickness to within 1% (<6 Å), not enough to explain a 12% difference in M_S_, which scales linearly with thickness (see the supplementary material).

In short, the FeRh layers are chemically and magnetically as near identical as possible in this extremely sensitive material system, which should be expected given their identical deposition conditions. On the other hand, changing the deposition temperature of the Ni layer has a dramatic effect on its crystallographic texture, as well as that of the W capping layer. XRD in Fig. 1(a) shows that when Ni was deposited at RT, it grew with a solely (002) texture, but when Ni was deposited at HT, the (002) orientation was partially suppressed in favor of a (022) texture. Similarly, W grew with a (002) texture on the HT Ni but showed a dominant (011) texture on top of the RT Ni. We attribute these texture changes of the Ni and W layers to the FeRh lattice expansion across the metamagnetic transition. Specifically, a closer look at the HT sample’s Ni (022)/W (002) texture reveals that these planes, which are parallel to the FeRh film, possess lower areal atomic density, and in the case of W, larger interatomic distances than the RT orientations that appear energetically favored by the expanded FeRh lattice.

**FIG. 2.**
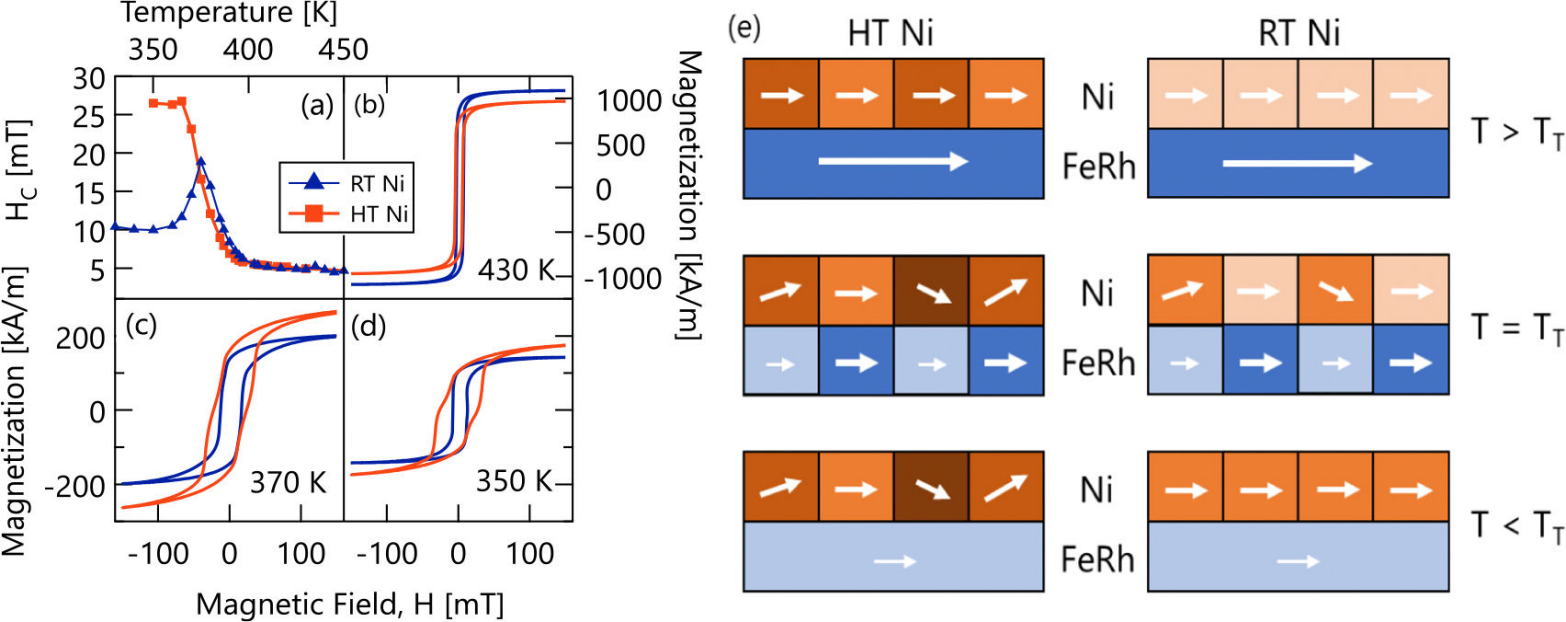
(a) Evolution of magnetic coercivity with temperature, extracted from Figs. 2(b) to 2(d). In-plane magnetization for FeRh/Ni bilayers at (b) 430 K, (c) 370 K, and (d) 350 K for RT Ni (blue triangles) and HT Ni (red squares). (e) Schematic illustrating how strain and crystal texture differences of the Ni film (coindicated by differing shades of orange) lead to enhanced coercivity in the HT sample but not the RT sample. Here, greater coercivity is depicted by the weaker magnetic alignment between the Ni grains arising from a combination of domain wall pinning and magnetic anisotropy differences between differently oriented Ni grains.

The different Ni deposition temperatures also change the field-dependent magnetization as shown in Fig. 2. Hysteresis loops at selected temperatures are shown in Figs. 2(b)–2(d). The values of the coercivity at different temperatures are extracted from the hysteresis loops and plotted in Fig. 2(a). Above 400 K, the values of the coercivity for both bilayers are controlled by the FeRh film and are almost identical in value near 5 mT. Upon cooling through T_T_, the magnetic behavior of the bilayers diverges. For the RT Ni sample, the coercivity increases, reaches a peak of 19 mT at 375 K, before decreasing back to 10 mT below the transition. The coercivity of the HT Ni sample closely tracks the RT Ni sample through 375 K, displays a small peak at approximately the same temperature as the RT Ni sample, but then plateaus to a value of 26 mT at 365 K. Note that while the data in Fig. 2 compares just two samples, additional samples grown under the same conditions replicate this behavior (see the supplementary material).

The switching behavior observed in the hysteresis loops is also affected by Ni deposition temperature. Above the transition, at 430 K, the FeRh dominates the magnetic behavior of the system, and a single switching in the hysteresis indicates that the Ni layer, regardless of deposition temperature, is exchange coupled to the FeRh film.

As the temperature decreases, a larger magnetic field must be applied for both RT and HT samples to close the loops [Fig. 2(c)], indicating a modified in-plane anisotropy below T_T_. In addition, a difference between the switching behavior of the RT and HT Ni samples emerges, as the sample cools through the magnetic transition. Beginning at 375 K, the HT Ni sample displays two-step switching that is not observed in the RT Ni samples. Examples are shown at 370 K and 350 K in Figs. 2(c) and 2(d), respectively. This behavior indicates that when the Ni is deposited on top of FeRh at HT (in the FM state), both layers are magnetically coupled above T_T_, but then act independently below T_T_, giving rise to the two-step switching event. Note that no exchange bias was observed in the bilayers, nor was it expected, given the compensated spin structure of the FeRh (001) planes at the Ni/FeRh interface.^33^

**FIG. 3.**
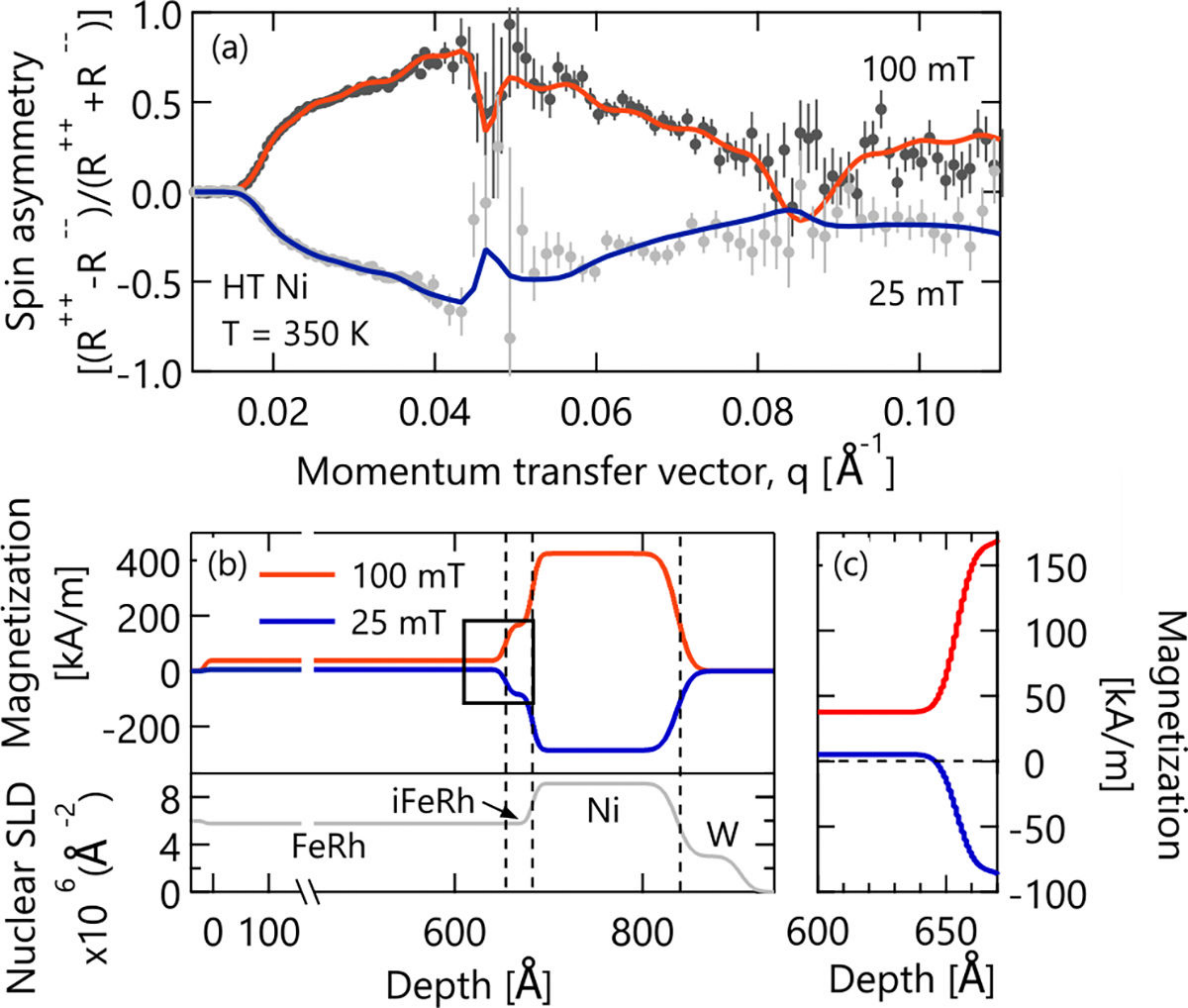
(a) Spin asymmetry data (points) and fits (lines) from PNR collected at 350 K on the HT Ni sample at 100 mT and 25 mT, the latter after cycling through negative saturation. Error bars are 1σ. (b) Magnetic and nuclear depth profiles corresponding to the fits in (a). (c) Enlarged area of the magnetic depth profile corresponding to gray box in (b) that highlights the independent switching of the bulk FeRh and Ni in the HT sample by showing that the bulk of the FeRh layer has a small, positive magnetization while Ni is still negatively polarized at 25 mT.

To better understand the double switching behavior in the HT sample, we performed PNR to resolve the layer-by-layer magnetic depth profile. Plotted in Fig. 3(a) is the 350 K spin asymmetry (SA), defined as the difference between up-up and down-down PNR channels, normalized by their sum, which highlights the magnetic contribution from scattering. For a completely nonmagnetic sample, the spin asymmetry would be zero. Our two PNR datasets essentially mirror one another across this SA = 0 divider, which indicates that some prominent feature of the magnetic depth profile has flipped sign.

The sample was modeled as three magnetic slabs: bulk FeRh, interfacial FeRh, and Ni. At 100 mT, all three slabs are aligned positive with respect to the field direction. At 25 mT, the bulk of the FeRh layer has followed the applied field and has a small, positive magnetization of 4.1 kA/m (95% confidence interval = 0.3–10.7 kA/m) as shown in Fig. 3(c). While FeRh is nominally AFM at 350 K, it is not uncommon to observe weak residual magnetization under applied field.^34^ Surprisingly, the Ni layer remains negatively polarized at +25 mT. It is this large, negative Ni magnetization that is responsible for the sign of the PNR spin asymmetry. Note that flipping the sign of the FeRh magnetization does not flip the spin asymmetry trend, and therefore these data unambiguously prove that it is the Ni layer in the HT Ni sample that has enhanced coercivity and switches second upon reversal.

In addition to the weak magnetization of the bulk FeRh, there is an interfacial FeRh layer about 3 nm thick that possesses stronger magnetism than the bulk of the layer below T_T_. The stabilization of the FM phase near interfaces is an extremely common behavior in the FeRh system and has been reported previously and attributed to a variety of origins.^35–37^ In our case, the common thickness of the interfacial magnetism seen in both the RT and HT samples and the different nuclear SLD structures we refine at those interfaces (see the supplementary material) suggests that the origin is not interdiffusion of Ni into the FeRh. However, Ni and Fe have very similar X-ray and neutron scattering lengths, which mean that interdiffusion of these species at the 1% level, a level sufficient to induce the observed increase in magnetization at the interface, is below our ability to confidently detect and, therefore, cannot be ruled out completely. Nevertheless, the precise origin of this interfacial magnetism does not impact our PNR analysis. Indeed, models without this interfacial FM region still capture the sign change of the SA in Fig. 3(a), which is only controlled by the sign of the Ni magnetization.

## DISCUSSION

Clearly, the Ni deposition temperature has a dramatic effect on the Ni coercivity at and below the FeRh transition. To understand why, we begin by considering the coercivity behavior of both samples at and above the midpoint of the FeRh magnetic transition. Well above T_T_, the thicker FM FeRh with larger M_S_ dominates the magnetic behavior of the whole system and the samples show similar coercivity values. Upon cooling, the coercivity of both RT and HT Ni samples peak at the midpoint of the FeRh magnetic transition [cf. Fig. 2(a)].A nearly identical behavior was observed in Ni/V_2_O_3_ bilayers upon crossing the V_2_O_3_ metal-to-insulator transition (MIT), which coincides with a first-order structural phase transition and a change in unit cell volume.^38^ In Ni/V_2_O_3_, it was concluded that the coexistence of metallic and insulating V_2_O_3_ phases with different in-plane lattice constants during the MIT creates magnetic domains with different strain states in the overlying Ni layer. Boundaries between these differently strained Ni regions act as pinning sites that inhibit domain wall motion during magnetic reversal and increase coercivity. The peak in coercivity at the midpoint of the transition corresponds to a maximum in the phase coexistence, when both metallic and insulating phases exist in equal populations and the phase boundary area is maximized. Given the similar shape of the coercivity behavior, and the direct analogy between the V_2_O_3_ MIT and FeRh metamagnetic transition, both accompanied by abrupt volume changes, we argue that the coercivity of the Ni/FeRh bilayers originates from precisely the same domain wall pinning mechanism due to the magnetic and structural phase coexistence within the Ni layer, during the first-order FeRh transition.

In contrast, below T_T_, the coercivity of the HT Ni/FeRh bilayer below T_T_ is 250% larger than the RT Ni value at the same temperature. We argue a similar link between structure and magnetization in the Ni layer is responsible. Specifically, when Ni is deposited at HT, the FeRh has a larger lattice parameter than it does at RT. As shown above, this creates a Ni film with multiple crystallographic orientations, which then become locked into different strain states when they cool below T_T_. Within this picture, schematically shown in Fig. 2(e), the enhanced coercivity could be explained by strain anisotropy and/or domain wall pinning between grains with different strain and orientation.^39,40^ However, our estimates of the strain induced anisotropy coming from magnetocrystalline and magnetoelastic terms suggest that these effects account for less than half of the coercivity change in our samples and instead point toward domain wall pinning as the dominant mechanism (see the supplementary material). Following from this argument, we would expect the opposite behavior for the Ni in the RT sample. Specifically, when Ni is grown at RT, it will be strained at higher temperatures and we would expect increased coercivity above T_T_. However, this effect is negated by the thicker FM FeRh that dominates the magnetic behavior above T_T_.

It is important to note that magnetic coupling between the FeRh and Ni is not the main driver of the coercivity enhancements we observe. Instead, the coercivity changes come about primarily from the crystallographic coupling of the FM film with a material that undergoes a structural phase transformation with a large change in unit cell volume and modifies the structure of the neighboring FM layer. Thus, the ability to lock-in large coercivities we have shown here should be generally applicable to a wide range of material systems. Of particular interest are materials with high magnetic anisotropy (e.g., FePt and CoPt), which are not only critical media for current perpendicular magnetic recording technologies but also have thinner domain walls that are more susceptible to pinning, and therefore, may exhibit even larger coercivity changes with this methodology. Previous work on FeRh/FePt bilayers has shown similar tunability of the coercivity via the metamagnetic FeRh transition^11^ but considered only the effect due to direct exchange coupling. FePt has a magnetostrictive coefficient comparable to Ni and is often deposited at an elevated temperature (748 K) to ensure a high-quality film, which would correspond to a compressive strain as in our HT samples. An enhanced effect can also be expected in giant magnetostrictive materials such as Terfenol-D (Tb_x_Dy_1−x_Fe_2_, x ≈ 0.3). While these materials have magnetic anisotropies similar to FePt, their magnetostrictive coefficients are orders of magnitude larger potentially leading to magnetoelastic energies comparable to the magnetic anisotropy and exchange coupling energies.^41^

## CONCLUSIONS

In conclusion, we have demonstrated that the coercivity of ferromagnetic Ni films can be thermally tuned by a factor of 5 over a 50 K temperature window by strain coupling to metamagnetic FeRh layers. Depositing the Ni layer at a temperature above the FeRh metamagnetic transition, where the FeRh has a larger unit cell volume relative to room temperature, alters the Ni crystallographic texture. Upon cooling through the metamagnetic transition, the FeRh lattice shrinks and strains the overlying Ni film. The weakly textured nature of the Ni film creates inhomogeneous strain gradients across grain boundaries of different orientations that pin domain walls and lead to the increased coercivity we measure. This effect relies on the structural coupling of a FM to a material with a large volume-change structural phase transition, and therefore provides a general mechanism for thermally modifying the coercivity of magnetic materials.

## Supplementary Material

Supp1

See the supplementary material for the replication of coercivity trends, reflectivity modeling, nickel film texture analysis, and strain induced magnetic anisotropy calculations.

## References

[1] Namai A, Yoshikiyo M, Yamada K, Sakurai S, Goto T, Yoshida T, Miyazaki T, Nakajima M, Suemoto T, Tokoro H, and Ohkoshi S, Nat. Commun 3, 1035 (2012). 22948817 10.1038/ncomms2038 PMC3658006

[2] Varghese B, Piramanayagam SN, Yang Y, Wong SK, Tan HK, Lee WK, and Okamoto I, J. Appl. Phys 115, 17B707 (2014).

[3] Baruth A, J. Appl. Phys 118, 093901 (2015).

[4] Nayak AK, Nicklas M, Chadov S, Khuntia P, Shekhar C, Kalache A, Baenitz M, Skourski Y, Guduru VK, Puri A, Zeitler U, Coey JMD, and Fesler C, Nat. Mater 14, 679 (2015). 25774953 10.1038/nmat4248

[5] Kryder MH, Gage EC, McDaniel TW, Challener WA, Rottmayer RE, Ju G, Hsia Y-T, and Erden MF, Proc. IEEE 96, 1810 (2008).

[6] Pan L. and Bogy DB, Nat. Photonics 3, 189 (2009).

[7] Ju G, Peng Y, Chang EKC, Ding Y, Wu AQ, Zhu X, Kubota Y, Klemmer TJ, Amini H, Gao L, Fan Z, Rausch T, Subedi P, Ma M, Kalarickal S, Rea CJ, Dimitrov DV, Huang P-W, Wang K, Chen X, Peng C, Chen W, Dykes JW, Seigler MA, Gage EC, Chantrell R, and Thiele J-U, IEEE Trans. Magn 51, 1 (2015).26203196

[8] Jubert P-O, Zong F, and Grobis MK, IEEE Trans. Magn 53, 1 (2016).

[9] Hu Y, Wu H, Meng Y, Wang Y, and Bogy B, J. Appl. Phys 123, 034303 (2018).

[10] Matlak J, Rismaniyazdi E, and Komvopoulos K, Sci. Rep 8, 9807 (2018). 29955072 10.1038/s41598-018-27688-4 PMC6023885

[11] Thiele J-U, Maat S, and Fullerton EE, Appl. Phys. Lett 82, 2859 (2003).

[12] Seki T, Utsumiya K, Nozaki Y, Imamura H, and Takanashi K, Nat. Commun 4, 1726 (2013). 23591893 10.1038/ncomms2737

[13] Kouvel JS and Hartelius CC, J. Appl. Phys 33, 1343 (1962).

[14] Kouvel JS, J. Appl. Phys 37, 1257 (1966).

[15] Maat S, Thiele J-U, and Fullerton EE, Phys. Rev. B 72, 214432 (2005).

[16] Cher KM, Zhou TJ, and Chen JS, IEEE Trans. Magn 47, 4033 (2011).

[17] Jiang M, Chen XZ, Zhou XJ, Wang YY, Pan F, and Song C, J. Cryst. Growth 438, 19 (2016).

[18] Lewis LH, Marrows CH, and Langridge S, J. Phys. D: Appl. Phys 49, 323002 (2016).

[19] Shirane G, Nathans R, and Chen CW, Phys. Rev 134, A1547 (1964).

[20] Bordel C, Juraszek J, Cooke DW, Baldasseroni C, Mankovsky S, Minar J, Ebert H, Moyerman S, Fullerton EE, and Hellman F, Phys. Rev. Lett 109, 117201 (2012). 23005667 10.1103/PhysRevLett.109.117201

[21] Stamm C, Thiele J-U, Kachel T, Radu I, Ramm P, Kosuth M, Minar J, Ebert H, Durr HA, Eberhardt W, and Back CH, Phys. Rev. B 77, 184401 (2008).

[22] Zakharov AI, Kadomtseva AM, Levitin RZ, and Ponyatovskii EG, Sov. Phys. JETP 19, 1348 (1964).

[23] Ibarra MR and Algarabel P, Phys. Rev. B 50, 4196 (1994). 10.1103/physrevb.50.4196 9976708

[24] de Vries MA, Loving M, Mihai AP, Lewis LH, Heiman D, and Marrows CH, New J. Phys 15, 013008 (2013). 10.3791/50603 PMC3938335 24145690

[25] van Driel J, Coehoorn R, Strijkers GJ, Bruck E, and de Boer FR, J. Appl. Phys 85, 1026 (1999).

[26] Suzuki I, Naito T, Itoh M, Sato T, and Taniyama T, J. Appl. Phys 109, 07C717 (2011).

[27] Annaorazov MP, Nikitin SA, Tyurin AL, Asatryan KA, and Dovletov A. Kh., J. Appl. Phys 79, 1689 (1996).

[28] Any mention of specific trade names and commercial products is for information only; it does not imply recommendation or endorsement by NIST.

[29] Kirby BJ, Kienzle PA, Maranville BB, Berk NF, Krycka J, Heinrich F, and Majkrzak CF, Curr. Opin. Colloid Interface Sci 17, 44 (2012).

[30] Maranville BB, Ratcliff II W, and Kienzle PA, J. Appl. Cryst 51, 1500 (2018).

[31] Maranville BB, Green A, and Kienzle PA, e-print arXiv:1801.04975 (2017).

[32] Inoue S, Ko HYY, and Suzuki T, IEEE Trans. Magn 44, 2875 (2008).

[33] Suzuki I, Hamasaki Y, Itoh M, and Taniyama T, Appl. Phys. Lett 105, 172401 (2014).

[34] Bennett SP, Wong AT, Glavic A, Urban C, Valmianski I, Biegalski MD, Christen HM, Ward TZ, and Lauter V, Sci. Rep 6, 22708 (2018). 10.1038/srep22708 PMC4778125 26940159

[35] Fan R, Kinane CJ, Charlton TR, Dorner R, Ali M, de Vries MA, Byrdson RMD, Marrows CH, Hickey BJ, Arena DA, Tanner BK, Nisbet G, and Langridge S, Phys. Rev. B 82, 184418 (2010).

[36] Baldasseroni C, Palsson GK, Bordel C, Valencia S, Unal AA, Kronast F, Nemsak S, Fadly CS, Borchers JA, Maranville BB, and Hellman F, J. Appl. Phys 115, 043919 (2014).

[37] Le Graet C, Charlton TR, McLaren M, Loving M, Morley SA, Kinane CJ, Byrdson RMD, Lewis LH, Langridge S, and Marrows CH, APL Mater. 3, 041802 (2015).

[38] de la Venta J, Wang S, Saerbeck T, Ramirez JG, Valmianski I, and Schuller IK, Appl. Phys. Lett 104, 062410 (2014).

[39] Livingston JD, J. Appl. Phys 52, 2544 (1981).

[40] Skomski R, J. Phys. Condens. Matter 15, R841 (2003).

[41] Goran E, Handbook of Giant Magnetostrictive Materials (Academic Press, San Diego, 2000).

