# Supplementary material for: Using structural phase transitions to enhance the coercivity of ferromagnetic films
Source: APL Mater. Author manuscript; Available in PMC 2026 Jun 5. (PMC13235367; doi:10.1063/1.5118893)
Supplement: Supp1 [file NIHMS1670527-supplement-Supp1.pdf]

SUPPLEMENTARY MATERIAL

R. F. Need et al., Using structural phase transitions to enhance the coercivity of ferromagnetic films

I. REPLICATION OF COERCIVITY BEHAVIOR

Figure 2(a) of the main text displays one of the main results of this work, namely, a dramatic change in the coercivity of the Ni layer when it is grown at high temperature (HT = 523 K) compared to when the Ni layer is grown at room temperature (RT = 300 K). To ensure this behavior is repeatable and intrinsic (i.e. not associated with extrinsic defects or sample-to-sample variation), we grew a second set of samples under identical conditions to those reported in the main text.

In Fig. S1, the temperature dependent coercivity behavior of these two replicant samples (#2) are plotted alongside the two samples from the main text (#1). While there is clearly a slight FeRh composition difference between the first and second set of films that manifests as a horizontal shift of the coercivity maximum (≈ metamagnetic transition temperature), within the HT and RT categories, the shape of the coercivity trends and even the quantitative value of the Ni coercivity at low temperatures are essentially identical. This indicates that the coercivity changes at below the transition temperature are primarily due to the Ni deposition temperature, and other factors, such as FeRh composition, have negligible effects on this behavior.

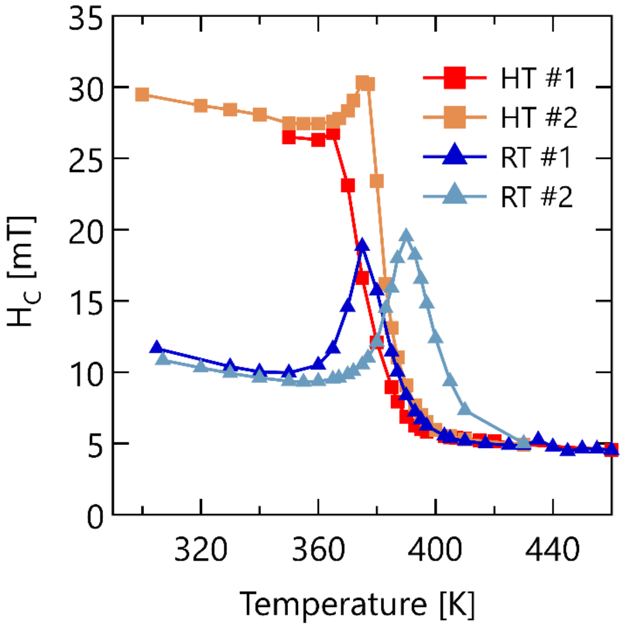

Figure S1: Coercivity extracted from magnetic hysteresis loops as a function of temperature highlighting the repeatability of the different behavior seen in samples where the Ni layer was deposited at HT (red) and RT (blue).

II. REFLECTIVITY DATA FITTING

To constrain the field-dependent polarized neutron reflectometry (H-dep PNR) models presented in Fig. 3 of the main text, we collected both X-ray reflectometry (XRR) and temperature-dependent PNR (T-dep PNR). These data were analyzed in a specific sequence (XRR → T-dep PNR → H-dep PNR) and the best models from the preceding fits used as initial input in modeling the subsequent data. The XRR data allowed us to determine an approximate chemical depth profile of our samples in the absence of the complicating magnetic signal in PNR. Similarly, by co-refining the T-dep PNR data set to a single nuclear (i.e. chemical) profile and only allowing the magnetic profile to change with temperature, the nuclear and magnetic structure of the samples can be disentangled.

All numerical refinements were done using a combination of two algorithms within the Refl1D package [1,2]. First, the DREAM algorithm was used to rapidly sample a wide parameter phase space and identify the general region of best fit. DREAM is a Markov chain Monte Carlo method with a differential evolution step generator. It explores parameter space using a random walk similar to simulated annealing algorithms, always accepting a better point in phase space but also accepting a worse point depending on how much worse and at what point in the regression it is. Regressions were run using approximately 1000 steps depending on the number of free parameters. Second, the Nelder-Mead simplex algorithm was used to find the absolute minimum within the region of phase space identified by the DREAM algorithm. The Nelder-Mead method, also sometimes referred to as the amoeba or polytope method, is a “downhill” algorithm that creates a simplex of $n+1$ points, where n is the dimensionality of the parameter space, then continually moves the simplex point where the goodness of fit is greatest eventually converging on the local, and hopefully global, minimum.

The raw PNR data, for both the T-dep and H-dep series, were first reduced by subtracting background scans from the signal, adjusting for polarization efficiencies, and correcting for the footprint of the incident beam.

Interfacial roughness of the slab layers within our scattering length density (SLD) profiles was modeled using the Nevot-Croce approximation, which treats the roughness as a Gaussian distribution. Roughness from each interface is propagated throughout the entire sample stack as described in Ref. [3] and implemented in the latest version of Refl1D.

Error bars plotted in the reflectometry data represent one standard deviation of the data. Error in the refined parameters (e.g. layer thicknesses, magnetization) are reported as 95 % confidence intervals calculated by the DREAM Monte Carlo algorithm in the Refl1D package as the range that contains 95 % of the accepted hops.

X-ray Reflectometry

The XRR data shown in Fig. S2 was collected in ambient conditions with a Rigaku SmartLab diffractometer equipped with a Cu Kα source, parallel beam optics, and a 0.5° Soller slit on the incident arm [4]. As can be seen from the best fit models in Fig. 2(b) and 2(d), both samples have

strikingly similar layer profiles. The major difference between the two is the presence of a thin region at the Ni/FeRh interface of the RT sample with low SLD. With only the XRR data, there are several possible explanations for this feature: Fe-rich FeRh, Ni diffusion into the FeRh, or a low-density Ni layer. We return to this feature below, using the results of the PNR data analysis to narrow down those possibilities. The other noteworthy difference is that the HT sample has a slightly rougher surface to its tungsten capping layer, which is the primary reason for the damped oscillations in the HT reflectivity relative to the RT sample.

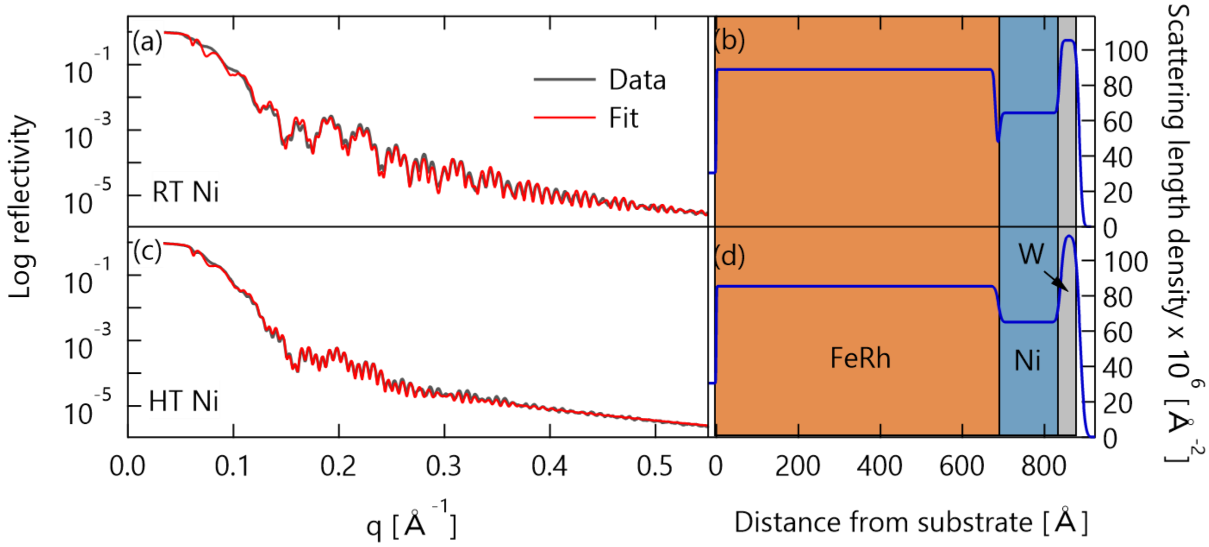

Figure S1: X-ray reflectivity of the RT (a) and HT (c) samples alongside the scattering length density models (b) and (d) that correspond to the fit curves.

Temperature-dependent Neutron Reflectometry

For both samples, PNR was collected at four temperatures across the FeRh metamagnetic transition temperatures (340 K, 375 K, 390 K, and 430 K). These four datasets were then co-refined to unique magnetic profiles but a common nuclear/chemical/structural profile. This approach provides greater confidence than individual fitting due to the increased ratio of data points to free parameters within the model. It is also a significantly quicker means deconvolving the nuclear and magnetic components of the PNR signal, relative to iteratively fitting the individual PNR curves. In short, co-refinement allows us to quickly and confidently determine a common nuclear structure profile and extract the changes to the magnetic depth profile across the metamagnetic transition.

Figure S3 shows the results of the co-refinement analysis for the RT sample. On the left are the PNR data and best fits, on the right are the corresponding nuclear and magnetic depth profiles. Looking first at the nuclear profile, we see that the layer thicknesses and general structure of the sample is very similar to that determined from our XRR analysis. Once again, there is a thin, low SLD layer at the Ni/FeRh interface. With this additional information from PNR, we can now eliminate a Fe-rich region and Ni diffusion into the FeRh as possible explanations, because both Fe and Ni have larger neutron scattering lengths than Rh and would thus push the SLD of that

region higher than the FeRh bulk. Therefore, the most likely explanation is a region of low-density Ni caused by the reduced adatom mobility when the film is deposited at room temperature.

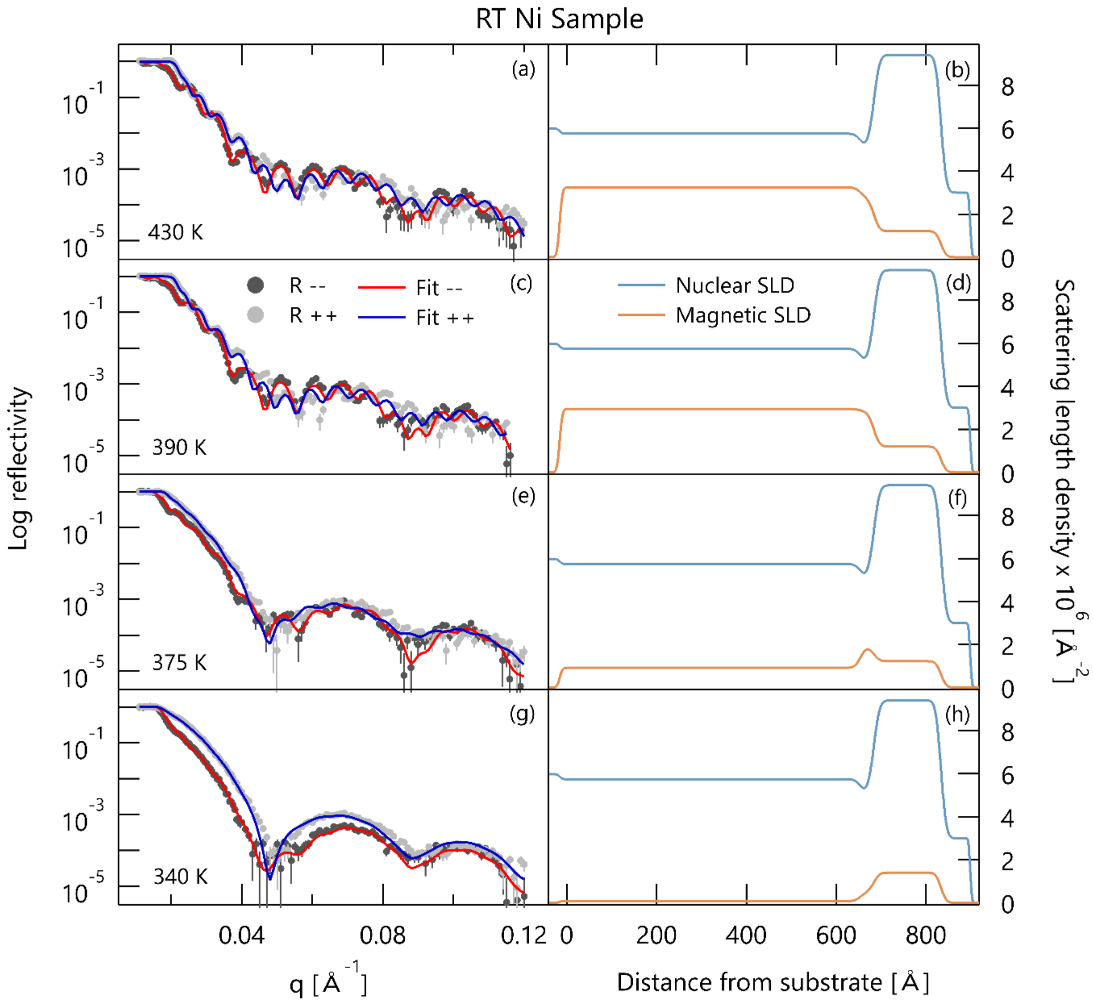

Figure S3: Temperature-dependent PNR data, fits, and models for the RT Ni sample. SLD models of the nuclear (chemical) and magnetic depth profiles are displayed to the right of their corresponding data.

The magnetic depth profiles for the RT sample evolve as expected from the bulk magnetometry. At high temperatures, the FeRh makes the largest contribution to the magnetic signal. As the temperature decreases through FeRh's metamagnetic transition, the FeRh magnetization decreases steadily while the Ni magnetization remains essentially unchanged. However, at 340 K, well below the transition, there remains a small FM component throughout the FeRh layer as well as an enhanced FM signal near the interface. This finding is consistent with several previous reports of residual FM in FeRh film and interfaces below the nominal magnetic temperature that have been attributed to interfacial strain or slight dopant interdiffusion from the neighboring layer [5-8]. It is important to note that multiple unique structural models with non-uniform magnetization

throughout the FeRh were tried, in which the magnetization at the FeRh/MgO substrate interface was allowed to be distinct from the bulk of the FeRh. However, each model with this freedom minimized to a best fit with a magnetically uniform profile near the substrate interface. While this result from any single model could be explained as the refinement algorithm getting trapped in an incorrect local minimum, multiple models displaying the same result provides significantly greater confidence in a sharp magnetic transition at the substrate.

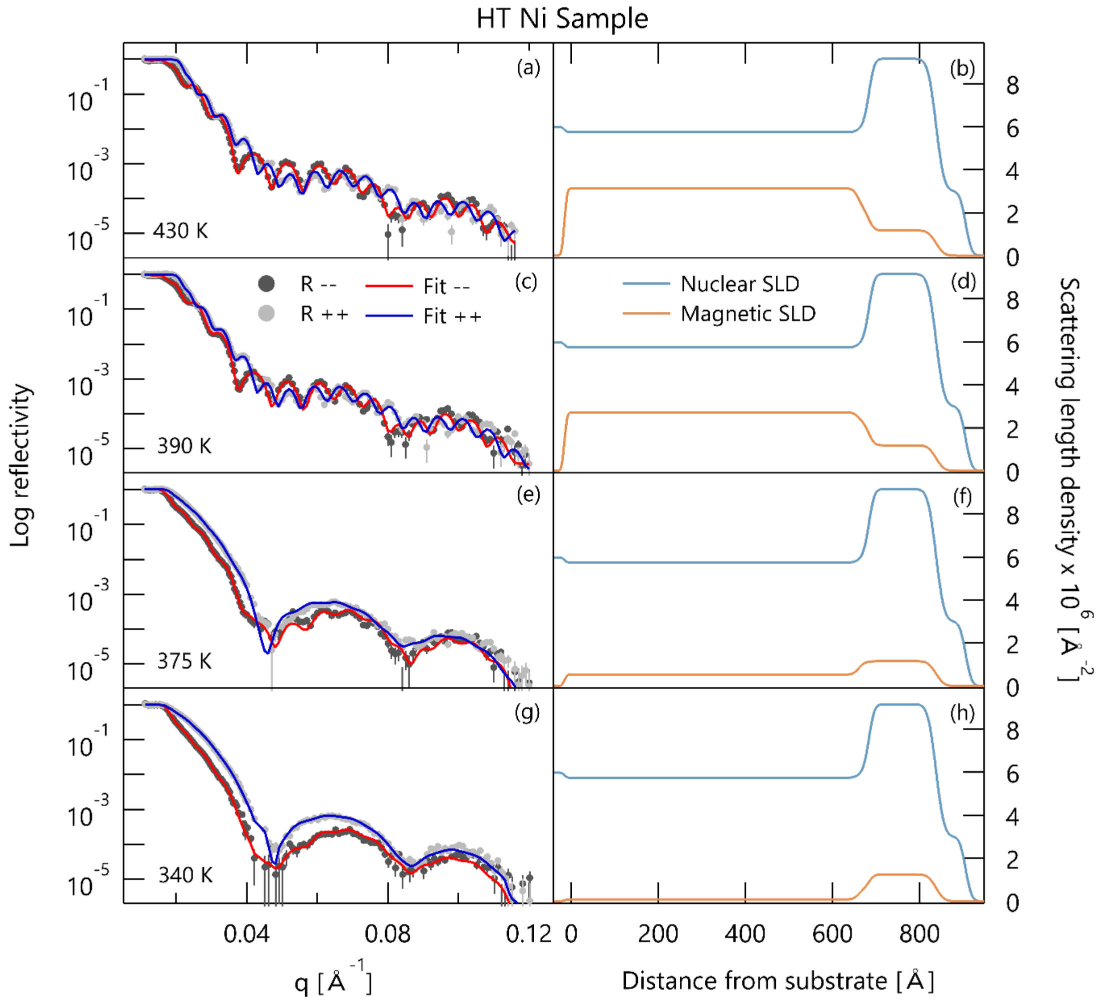

Figure S4: Temperature-dependent PNR data, fits, and models for the HT Ni sample. SLD models of the nuclear (chemical) and magnetic depth profiles are displayed to the right of their corresponding data.

Co-refining the T-dep PNR data for the HT sample yields very similar results to the RT sample. First, the nuclear/structural depth profile is nearly identical to that determined from XRR, and still shows a rougher tungsten surface compared to the RT sample along with a smoother transition across the Ni/FeRh interface. Regarding the magnetic profiles, as seen in the RT sample, there is a slight enhancement of the magnetization in the FeRh immediately adjacent to the Ni interface

that is subtler than seen in the RT sample. Otherwise, the magnetic behavior of the HT sample follows the same temperature trends as the RT sample and bulk magnetometry.

III. NICKEL FILM TEXTURE

While Ni films commonly grow with a (111) texture [9], the only obvious Ni peaks in our XRD are the (002) and (022) as shown in Fig. S5. A close examination of the region surrounding the MgO (002) shown in Fig. S6(a) reveals that the unstrained Ni (111) reflection, which would occur at $2\Theta = 44.5°$, is notably absence in both films. There is a difference between the two samples at smaller angles with a shoulder near 43.5°, which would correspond to about 2% out-of-plane expansion of the Ni (111) planes. However, we do not believe that this shoulder corresponds to Ni (111) for a few reasons. First, the shoulder is present at the same position in both samples, so the hypothetical strain cannot be explained by the FeRh structural transition, which would only affect the film grown at HT in our XRD measurements taken at RT. Second, the Ni reflections we do observe (i.e. (002) and (022)) occur very near the angles expected for a bulk-like, unstrained Ni structure and it is therefore difficult to understand why only Ni (111) would be strained. Third, when the difference between the HT and RT XRD pattern is plotted, the result looks more like a subtle difference in the MgO substrates than a small film peak beneath the MgO shoulder. Altogether, this suggests that there is little-to-no Ni (111) texture present in either of our films.

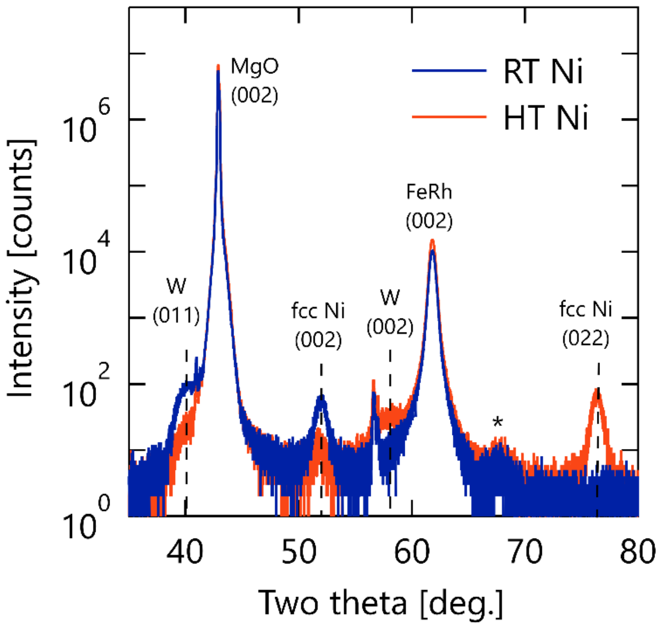

Figure S5: Room temperature X-ray diffraction of the RT and HT samples overlaid to highlight changes in Ni and W texture between the two samples.

Quantitative attempts to fit a Ni (111) reflection to the HT Ni data are shown in Fig. S6(b) and 6(c). While the addition of a peak at 43.5° in Fig. S6(b) does improve the visual fit on the righthand side of the MgO reflections, the improvement in $\chi 2$ amounts to less than 0.3% because

of the large difference in intensity between the shoulder and the MgO peaks, and therefore it is difficult to make any concrete conclusions from this analysis.

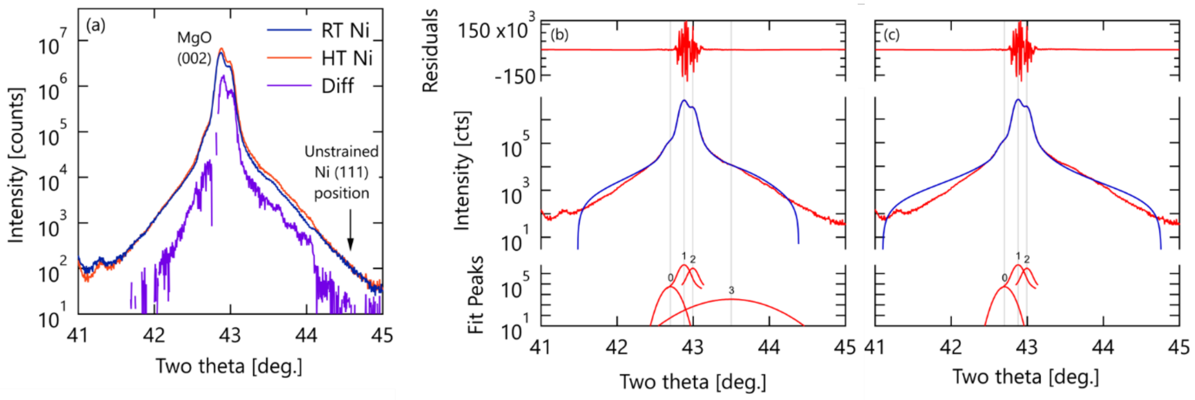

Figure S6: (a) X-ray diffraction of the MgO (002) showing an absence of the Ni (111) reflection at the expected position. (b) Fitting analysis of peaks in the vicinity of MgO (002) for the HT Ni sample.

IV. STRAIN INDUCED MAGNETIC ANISOTROPY CALCULATIONS

In order to better understand the origin of the low temperature coercivity differences between RT and HT samples, the effect of strain induced magnetic anisotropy on the different films was estimated. A quantitative estimate of the strain induced anisotropy can be obtained by considering the various contributions to the system's free energy, which can be given approximately as:

$$\mathrm{E_{tot} = E_0 + E_{mc} + E_{me}}$$

where $\mathrm{E_{tot}}$ is the total energy required for saturation [10]. The energy required to saturate the unstrained film can be written as $\mathrm{E_0} = \frac{1}{2}\mathrm{H_0 M_S}$, with $\mathrm{H_0}$ the magnetic field required to saturate the unstrained film [10]. $\mathrm{E_{mc}}$ and $\mathrm{E_{me}}$ represent the magnetocrystalline and magnetoelastic contributions to the free energy, respectively.

For a cubic crystal, the magnetocrystalline anisotropy can written as:

$$\mathrm{E_{mc}} = \mathrm{K_1}(\alpha_1^2 + \alpha_2^2 + \alpha_3^2) + \mathrm{K_2}(\alpha_1^2\alpha_2^2\alpha_3^2)$$

where $\mathrm{K_1}$ and $\mathrm{K_2}$ are the typical anisotropy constants for Ni and the α_i are the direction cosines of the magnetization [11]. For the RT sample, where the Ni in an (00L) orientation, this calculation results in-plane anisotropy values of $\mathrm{E_{mc}^{110} - E_{mc}^{001}} = -1370\frac{\mathrm{J}}{\mathrm{m^3}}$. A similar calculation can be performed on the (0LL) Ni of the HT sample after rotating into the crystal reference frame with a 3x3 rotation matrix, assuming the film is elastically isotropic and any shear strains are negligible [12]. Under these assumptions, we calculate $\mathrm{E_{mc}^{\sqrt{2}11} - E_{mc}^{100}} = -2817\frac{\mathrm{J}}{\mathrm{m^3}}$, where the *hkl* superscripts refer to the crystal coordinate system for both systems (i.e. $\sqrt{2}11$ with the Ni layer oriented such that (022) is out-of-plane corresponds to the (110) plane in the lab frame).

Estimates for the effective field were made using the relation:

$$H_{eff} = \frac{2\Delta E}{M_S}$$

which yields effective field values of $H_{eff} \cong 5.7$ mT and $H_{eff} \cong 11.7$ mT for the RT and HT samples, respectively. The 6 mT difference between the two samples indicates that while the magnetocrystalline anisotropy certainly plays a role, it isn't primarily responsible for the difference 15 mT between the samples.

The other contribution to the free energy is the magnetoelastic energy. The out-of-plane strain can be estimated using the relation between the lattice parameter of the Ni film obtained from XRD compared to bulk Ni ($a = 3.5238$ Å [13]).

$$\epsilon_{zz} = \frac{a_{film} - a_{bulk}}{a_{bulk}}$$

In the RT case, there is no contribution to the in-plane anisotropy despite a strain of $\epsilon_{zz} = -0.24\%$ as strain is isotropic in-plane. However, there is a nonzero contribution to the HT sample due to the way the different orientation acts under biaxial strain. For the HT sample, a very slight out-of-plane compressive strain $\epsilon_{zz} = -0.0046\%$ is found, corresponding to a nearly relaxed film. The proper in-plane strain can be found by performing a similarity transformation into the crystal frame and minimizing the elastic and magnetoelastic energies. The appropriately transformed difference in free energies yields $E_{me}^{100} - E_{me}^{001} = -412\,\frac{J}{m^3}$ and corresponds to an effective field $H_{eff} \cong$ 1.8 mT. While not trivial, this is an order of magnitude too small to explain the difference between RT and HT films shown in Fig. 2(a).

However, the direct magnetoelastic calculations here do not account for alternative effective coupling mechanisms, such as domain wall pinning or magnetic coupling to any remnant FM phase at the interface. The strain relationship between the Ni and FeRh is also complicated by the large ($> 10\%$) lattice mismatch between the two for both Ni orientations, rather than simple epitaxy. This likely leads to some nontrivial depth dependence to the strain as well as the development of dislocations to relieve the stress, which can act as additional domain wall pinning centers. In summary, our estimations for strain induced anisotropy show that while the effect is not negligible, it is not large enough to account for the contrasting coercivity behavior between the RT and HT samples.

REFERENCES

[1] B. J. Kirby, P. A. Kienzle, B. B. Maranville, N. F. Berk, J. Krycka, F. Heinrich, and C. F. Majkrzak, Curr. Opin. Colloid Interface Sci. **17**, 44 (2012).

[2] B. B. Maranville, W. Ratcliff II, and P. A. Kienzle, J. Appl. Cryst. **51**, 1500-1506 (2018).

[3] B.B. Maranville, A. Green, P.A. Kienzle. arXiv. 1801.04975 (2018).

[4] Any mention of specific trade names and commercial products is for information only; it does not imply recommendation or endorsement by NIST.

[5] R. Fan, C. J. Kinane, T. R. Charlton, R. Dorner, M. Ali, M. A. de Vries, R. M. D. Brydson, C. H. Marrows, B. J. Hickey, D. A. Arena, B. K. Tanner, G. Nisbet, and S. Langridge, Phys. Rev. B **82**, 184418 (2010).

[6] K.M. Cher, T.J. Zhou, and J.S. Chen, IEEE Trans. Magn. **47**, 4033 (2011).

[7] C. Baldasseroni, G. K. Pálsson, C. Bordel, S. Valencia, A. A. Unal, F. Kronast, S. Nemsak, C. S. Fadley, J. A. Borchers, B. B. Maranville, and F. Hellman, J. Appl. Phys. **115**, 043919 (2014).

[8] C. Le Graët, T. R. Charlton, M. McLaren, M. Loving, S. A. Morley, C. J. Kinane, R. M. D. Brydson, L. H. Lewis, S. Langridge, and C. H. Marrows, APL Mater. **3**, 041802 (2015).

[9] M. S. Miller, F. E. Stageberg, Y. M. Chow, K. Rook, and L. A. Heuer, J. Appl. Phys. 75, 5779 (1994).

[10] J. Cui, J. L. Hockel, P. K. Nordeen, D. M. Pisani, C.-Y. Liang, C. P. Carman, and C. S. Lynch. Appl. Phys. Lett. 103, 232905 (2013).

[11] C. Kittel. Rev. Mod. Phys. 21, 541 (1949).

[12] D. Sander. Rep. Prog. Phys. 62, 809 (1999).

[13] Y. Tan, K. Liang, Z. Mei, P. Zhou, Y. Liu, Y. Qi, Z. Ma, and T. Zhang. Ceram. Int. 44, 5564 (2018).
